# The Relationships Between Radiation Dosage and Long-term Swallowing Kinematics and Timing in Nasopharyngeal Carcinoma Survivors

**DOI:** 10.1007/s00455-021-10311-6

**Published:** 2021-04-28

**Authors:** Dai Pu, Victor H. F. Lee, Karen M. K. Chan, Margaret T. Y. Yuen, Harry Quon, Raymond K. Y. Tsang

**Affiliations:** 1 Department of Surgery, LKS Faculty of Medicine, The University of Hong Kong, Hong Kong, China; 2 Swallowing Research Laboratory, Faculty of Education, The University of Hong Kong, Hong Kong, China; 3 School of Primary and Allied Health Care, Faculty of Medicine, Nursing and Health Sciences, Monash University, Frankston, Australia; 4 Department of Clinical Oncology, LKS Faculty of Medicine, The University of Hong Kong, Hong Kong, China; 5 Department of Radiation Oncology and Molecular Radiation Sciences, Oncology and Otolaryngology and Head and Neck Surgery, Johns Hopkins University, Baltimore, USA; 6 Department of Surgery and Department of ENT, Queen Mary Hospital, 102 Pokfulam Road, Hong Kong, China

**Keywords:** Nasopharyngeal carcinoma, Head and neck cancer, Intensity-modulated radiotherapy, Radiation dosage, Deglutition disorders, Deglutition, Biomechanical phenomena, Fluoroscopy

## Abstract

This study aimed to investigate the relationship between intensity-modulated radiation therapy (IMRT) dosimetry and swallowing kinematic and timing measures. Thirteen kinematic and timing measures of swallowing from videofluoroscopic analysis were used as outcome measures to reflect swallowing function. IMRT dosimetry was accessed for thirteen swallowing-related structures. A cohort of 44 nasopharyngeal carcinoma (NPC) survivors at least 3 years post-IMRT were recruited. The cohort had a mean age of 53.2 ± 11.9 years, 77.3% of whom were male. There was an average of 68.24 ± 14.15 months since end of IMRT; 41 (93.2%) had undergone concurrent chemotherapy. For displacement measures, female sex and higher doses to the cricopharyngeus, glottic larynx, and base of tongue were associated with reduced hyolaryngeal excursion and pharyngeal constriction, and more residue. For timing measures, higher dose to the genioglossus was associated with reduced processing time at all stages of the swallow. The inferior pharyngeal constrictor emerged with a distinctly different pattern of association with mean radiation dosage compared to other structures. Greater changes to swallowing kinematics and timing were observed for pudding thick consistency than thin liquid. Increasing radiation dosage to swallowing-related structures is associated with reduced swallowing kinematics. However, not all structures are affected the same way, therefore organ sparing during treatment planning for IMRT needs to consider function rather than focusing on select muscles. Dose-response relationships should be investigated with a comprehensive set of swallowing structures to capture the holistic process of swallowing.

## Introduction

Nasopharyngeal carcinoma (NPC) is endemic in Southern China and South-East Asia [1]. Treatment for NPC has been improving for the past several decades. Advancements in treatment technology and practice, such as concomitant chemotherapy [2] and intensity-modulated radiation therapy (IMRT) [3], have resulted in significant improvements in survival [1, 4]. The emerging issues that accompany these advancements now point towards radiation-associated late sequelae [5].

One late but salient sequela of NPC treatment is dysphagia, abnormality of the process of swallowing. It can manifest as subjective reports of swallowing difficulties and discomfort, and abnormalities in swallowing physiology via instrumental examinations such as endoscopy or videofluoroscopy; the latter of which can provide insights into changes to swallowing function as a result of cancer treatment. Dysphagia can pose a negative impact on the quality-of-life of NPC survivors [6]. A study that examined a group of head and neck cancer survivors, more than half of whom had been diagnosed with NPC, demonstrated that experiences around meals were intrinsically altered following treatment, and accordingly the life and social events that involved eating and drinking were negatively affected [7]. Subjective dysphagia can be observed in up to 84% of NPC survivors [6, 8], and instrumental examination using endoscopy or fluoroscopy have found even higher rates for specific functional abnormalities (e.g. failed airway protection, food residue in the pharynx, prolonged processing time) [8, 9] that worsen as time progresses [10].

A recent review found impairments at the oral, pharyngeal, and oesophageal stages of deglutition in NPC survivors, with impairments in the latter two stages being the most prevalent [11]. This is likely due to the fact that targets for irradiation in NPC are primarily dysphagia/aspiration-related structures (DARS) that affect these two stages. IMRT allows radiation to focus on tumour sites while minimising the dosage administered to the surrounding healthy tissues that may be DARS. A systematic review of dose-volume correlates for structures related to late dysphagia identified the pharyngeal constrictors [12], but more recent findings suggest that the mean dose to the floor of the mouth and the geniohyoid may also be pertinent [13]. NPC-specific DARS have been initially identified as the inferior and superior pharyngeal constrictors [11]. How exactly irradiation to these and other potential DARS leads to the specific functional abnormalities documented is not well explored. Better understanding of how swallowing is affected can offer potential solutions to rehabilitation and long-term management of dysphagia in NPC survivors.

### Objectives

This study aimed to investigate the relationship between radiation dosimetric parameters of swallowing-related structures with IMRT and long-term swallowing kinematics and timing in nasopharyngeal carcinoma survivors.

## Materials and Methods

### Participants

This was a cross-sectional study. NPC survivors who received their curative intent treatment with IMRT with or without concurrent/induction/adjuvant chemotherapy for more than 3 years prior to recruitment were eligible for the study. Participants were recruited from an outpatient ear, nose, and throat clinic at a tertiary hospital. The following exclusion criteria were used to limit additional influences on swallowing function:

1. persistent or recurrent NPC;
2. received more than one course of external radiotherapy or brachytherapy;
3. surgery to the head and neck area, except excision of skin lesions;
4. required enteral tube support for nutrition due to severe aspiration;
5. required tracheostomy;
6. history of neurological disease which would likely affect swallowing function;
7. dysfunction of cranial nerves IX, X, XI and XII;
8. no available IMRT planning data;
9. unable to provide consent or aged 18 years and under.

All participants provided written consent to complete swallowing assessments and allowed their hospital records to be accessed for research. This study was approved by the Institutional Review Board for the University of Hong Kong/Hospital Authority Hong Kong West Cluster (UW 15-140).

### IMRT Dosimetry

Computer tomography (CT) images used in IMRT planning for all recruited NPC patients in the software 360 Oncology© (Varian) were used for delineation of swallowing-related structures with Eclipse Treatment Planning System version 15.0 (Varian Medical Systems, Palo Alto, CA, USA). The total global radiation dosimetry administered to each participant during treatment was obtained. The second author, an oncologist, completed 30% of the contouring of structures, and the remainder 70% was completed by the first author after training by the oncologist. Contouring was completed according to guidelines [13–15] for the following structures:

- Oral cavity
- Base of tongue
- Pharyngeal constrictors: superior (SPC), middle (MPC), and inferior (IPC)
- Cricopharyngeus
- Larynx: glottic and supraglottic
- Suprahyoid: anterior digastric, genioglossus, geniohyoid, hyoglossus, and mylohyoid

The median dose of radiation (in Gy) to each structure was recorded.

### Swallowing Kinematics

Each participant completed a single session of videofluoroscopic study of swallowing (VFSS). Each VFSS session consisted of the participant swallowing 10 ml trials of thin liquid and pudding, 3 trials of each texture were swallowed. Each swallow was analysed using coordinate mapping with the software ImageJ [16] following preparation [17]. The cranial base, mandible, and C1, C2 and C4 of the vertebrae were fixed reference points. Five time-points were recorded: (1) oral hold (one frame before posterior transfer of bolus begins), (2) bolus head crosses the ramus of the mandible, (3) first frame of the antero-superior movement of the hyoid associated with swallow reflex initiation, (4) frame of maximum hyoid excursion, and (5) closure of the upper esophageal sphincter. The esophageal sphincter, cricoid and hyoid cartilages, and the pit of the valleculae were tracked as dynamic points; changes in displacement between time-points (1) and (3) were recorded to document hyolaryngeal movement during the swallow.

Pharyngeal space was outlined at time-points (1) and (3) to calculate pharyngeal constriction ratio. Areas of residue were calculated as a ratio of the total areas of the valleculae and pyriform sinuses. A coin with a diameter of 22.5 mm was used as scalar to measure length of displacement. All displacements were reported in centimetres. All time measures were reported as seconds. Following these methods, each swallow produced 13 kinematic measures (Table 1).

**Table 1:** Kinematic and timing measures of swallowing captured

Displacement measures	Anterior hyoid movement [18]
	Superior hyoid movement [18]
	Superior laryngeal movement [19]
	Hyolaryngeal approximation [20]
	Laryngeal elevation to cranial base [17]
	Pharyngeal shortening [17]
Spatial ratios	Tongue base retraction [21]
	Pharyngeal constriction ratio [22]
	Vallecular residue ratio [23]
	Pyriform sinus residue ratio [23]
Timing measures	Oral transit time [24]
	Stage transition time [24]
	Pharyngeal transit time [24]

Three swallows of each texture were conducted, the kinematic measures for each consistency were averaged and the mean used for analysis.

### Statistical Analysis

With kinematic measures as independent outcome variables, backward stepwise multiple linear regression models were constructed using dosimetry to the swallowing-related structures and patient demographic and treatment variables. Variables were considered statistically significant at *p* < 0.05.

## Results

A total of 44 NPC survivors underwent VFSS and their hospital records were retrieved to obtain demographic information and NPC treatment history (Table 2). The total median radiation dosage delivered to each participant was 66 Gy (range 66–76). The dosage of radiation for each swallowing-related structure was extracted (Table 3).

**Table 2:** Descriptive information of the 44 NPC survivors who participated in this study

Age	Mean = 53.2 years, range 25–75, standard deviation = 11.9
Sex	
Male	34 (77.3%)
Female	10 (22.7%)
Tumour	
T1	13 (29.5%)
T2	9 (20.5%)
T3	20 (45.5%)
T4	2 (4.5%)
Node	
N0	9 (20.5%)
N1	11 (25%)
N2	21 (47.7%)
N3	3 (6.8%)
Metastasis	0
Concurrent chemotherapy	41 (93.2%)
Length of IMRT	Mean = 44.7 days, range 40–49, standard deviation = 1.75
Total Dose Delivered (Gy)	Median = 66, range 66–76
Time Since Treatment	Mean = 68.24 months, range 42.90–94.98, standard deviation = 14.15

**Table 3:** Summary of radiation dosimetry (Gy) of the swallowing-related structures

	Mean ± Standard Deviation	Range (minimum–maximum)
Oral cavity	42.306 ± 10.643	0.005–54.160
Base of tongue	49.841 ± 12.820	0.067–75.075
Superior pharyngeal constrictor	62.412 ± 14.735	0.128–72.658
Middle pharyngeal constrictor	48.037 ± 11.829	0.444–67.862
Inferior pharyngeal constrictor	47.580 ± 11.697	0.881–61.541
Cricopharyngeus	47.251 ± 11.196	5.236–61.130
Glottic larynx	43.314 ± 10.316	0.743–58.281
Supraglottic larynx	45.153 ± 11.135	0.343–63.666
Anterior digastric	38.292 ± 13.904	0.021–69.318
Genioglossus	37.943 ± 12.837	0.006–54.225
Geniohyoid	38.612 ± 14.056	0.042–70.360
Hyoglossus	50.772 ± 16.996	0.008–68.709
Mylohyoid	40.558 ± 14.251	0.015–69.400

A total of 264 swallows were recorded for the 44 participants. Due to technological error, one participant’s pudding thick swallow trials were unable to be analysed, bringing the number of swallows analysed to 261. With two textures per participant and 13 mean kinematic measures for each texture, a total of 3393 kinematic measures were extracted. The mean for each kinematic measure across 3 swallows of each texture was calculated, 1131 mean kinematic measures were used for analysis (Table 4).

**Table 4:** Kinematic (cm) and timing (seconds) measures of swallowing for thin and pudding textures

	Thin liquid (mean ± standard deviation)	Pudding (mean ± standard deviation)	Difference between consistencies (paired sample *T* test)
Anterior hyoid movement	0.529 ± 0.441	0.602 ± 0.405	*p* = 0.089
Superior hyoid movement	0.169 ± 0.096	0.198 ± 0.088	*p* = 0.025
Superior laryngeal movement	0.637 ± 0.525	0.620 ± 0.514	*p* = 0.84
Hyolaryngeal approximation	0.200 ± 0.121	0.200 ± 0.144	*p* = 0.996
Laryngeal elevation to cranial base	1.522 ± 1.188	1.549 ± 0.848	*p* = 0.0839
Pharyngeal shortening	0.460 ± 0.182	0.511 ± 0.178	*p* = 0.04
Base of tongue retraction	0.759 ± 0.167	0.793 ± 0.195	*p* = 0.351
Pharyngeal Constriction ratio	0.065 ± 0.049	0.137 ± 0.130	*p* < 0.001
Valleculae residue ratio	0.131 ± 0.169	0.359 ± 0.323	*p* < 0.001
Pyriform residue ratio	0.094 ± 0.139	0.121 ± 0.207	*p* = 0.293
Oral stage time	0.713 ± 0.856	1.293 ± 1.721	*p* = 0.011
Stage transition time	0.097 ± 0.331	0.198 ± 0.458	*p* = 0.187
Pharyngeal stage time	0.677 ± 0.580	0.837 ± 0.748	*p* = 0.01

For each measure of swallowing function for each texture, different variables (radiation dosage to all the considered key structures, cancer stage, and demographics) were analysed statistically for significant associations in backward stepwise multiple linear regression (Table 5). Current chemotherapy was tested in initial exploratory analysis for associations with swallowing outcomes, but it was not found to be significant for this sample. The *p* values and co-efficient (*B*) for each variable is reported in Table 5. A *p* value of less than 0.05 indicated a significant association; the B indicated the nature of the association (i.e. for one increase in the variable, a negative or positive change in the swallowing function is indicated by the value of *B*). Results showed a number of structures and variables that are associated with changes in specific measures of swallowing function. For displacement measures, most variables showed a negative relationship (negative B), meaning that increasing dosage to these structures was associated with reduced displacement, denoting worse swallowing performance. For residue measures, most variables showed positive relationships (positive B), indicating that increasing dosage led to more residue. For timing measures, mixed results were found with increasing dosage to some structures leading to increased processing time (positive B) and some to decreased processing time (negative B).

**Table 5:** Multiple linear regression outcomes for dosimetry to swallowing-related structures and kinematic and timing measures of swallowing

	Anterior hyoid movement		Superior hyoid movement		Superior laryngeal movement		Hyolaryngeal approximation		Laryngeal elevation		Pharyngeal shortening		Tongue base retraction	
Texture	Thin	Puree	Thin	Puree	Thin	Puree	Thin	Puree	Thin	Puree	Thin	Puree	Thin	Puree
Adjusted R squared	0.252	0.102	0.447	0.317	0	0.428	0	0.447	0.164	0.241	0	0.207	0.176	0.312
Oral cavity				*p* = 0.058 *B* = − 0.493		*p* = 0.013 *B* = − 0.68		*p* = 0.005 *B* = − 0.872						
Base of tongue						*p* = 0.059 *B* = 0.449		*p* = 0.01 *B* = 0.639						*p* = 0.04 *B* = 0.62
Middle pharyngeal constrictor										*p* = 0.056 *B* = − 1.033		*p* = 0.008 *B* = − 0.86		
Inferior pharyngeal constrictor	*p* = 0.001 *B* = 1.207	*p* = 0.014 *B* = 0.974	*p* < 0.001 *B* = 1.556	*p* = 0.002 *B* = 1.032		*p* = 0.004 *B* = 0.878		*p* = 0.038 *B* = 0.614		*p* = − 0.12 *B* = 1.123		*p* = 0.053 *B* = 0.681		
Cricopharyngeus				*p* = 0.014 *B* = − 0.713		*p* = 0.001 *B* = − 0.869		*p* = 0.011 *B* = − 0.676						*p* = 0.008 *B* = − 0.749
Glottic larynx	*p* = 0.001 *B* = − 1.326	*p* = 0.016 *B* = − 0.944	*p* < 0.001 *B* = − 1.721						*p* = 0.037 *B* = − 0.646	*p* = 0.01 *B* = − 1.203			*p* = 0.05 *B* = 0.332	*p* = 0.012 *B* = 1.034
Supraglottic larynx										*p* = 0.039 *B* = 1.024				
Anterior digastric				*p* = 0.003 *B* = 1.153				*p* = 0.039 *B* = − 0.707					*p* = 0.035 *B* = 0.899	
Genioglossus														*p* = 0.002 *B* = − 1.318
Geniohyoid									*p* = 0.003 *B* = − 1.505				*p* = 0.024 *B* = − 0.966	
Hyoglossus	*p* = 0.019 *B* = 0.591		*p* = 0.001 *B* = 1.035							*p* = 0.034 *B* = 0.769				
Mylohyoid			*p* = 0.003 *B* = − 0.806	*p* = 0.019 *B* = − 0.902				*p* = 0.035 *B* = 0.764	*p* = 0.003 *B* = 1.55	*p* = 0.017 *B* = − 0.855				*p* = 0.006 *B* = 1.134
Age			*p* = 0.015 *B* = − 0.296											
Sex						*p* = 0.001 *B* = − 0.437		*p* = 0.002 *B* = − 0.42		*p* = 0.014 *B* = − 0.375		*p* = 0.015 *B* = − 0.351		
IMRT length			*p* = 0.001 *B* = 0.443										*p* = 0.011 *B* = 0.412	
Days since IMRT			*p* = 0.055 *B* = 0.251											

	Pharyngeal constriction ratio		Valleculae Residue Ratio		Pyriform Residue Ratio		Oral Stage Time		Stage Transition Time		Pharyngeal Stage Time	
Texture	Thin	Puree	Thin	Puree	Thin	Puree	Thin	Puree	Thin	Puree	Thin	Puree
Adjusted R squared	0.506	0.442	0.098	0.335	0.289	0.300	0.109	0.331	0	0.261	0	0.117
Oral cavity		*p* = 0.009 *B* = 0.752		*p* = 0.003 *B* = − 0.903						*p* = 0.019 *B* = 0.443		
Base of tongue				*p* = 0.008 *B* = 0.68	*p* = 0.001 *B* = 0.929	*p* = 0.004 *B* = 0.841						
Superior pharyngeal constrictor					*p* = 0.001 *B* = − 1.27	*p* = 0.02 *B* = − 0.696		*p* = 0.014 *B* = − 0.801				
Middle pharyngeal constrictor		*p* = 0.01 *B* = 1.258						*p* = 0.006 *B* = 0.883				
Inferior pharyngeal constrictor	*p* = 0.028 *B* = − 0.75	*p* < 0.001 *B* = − 1.71										
Glottic larynx	*p* = 036 *B* = 0.025	*p* = 0.015 *B* = 0.974	*p* = 0.047 *B* = 0.297									
Supraglottic larynx												*p* = 0.046 *B* = 0.361
Anterior digastric					*p* = 0.023 *B* = 0.384							
Genioglossus		*p* = 0.006 *B* = − 1.02				*p* = 0.033 *B* = − 0.75	*p* = 0.016 *B* = − 0.753	*p* < 0.001 *B* = − 1.575		*p* = 0.003 *B* = − 0.58		*p* = 0.011 *B* = − 0.469
Geniohyoid	*p* = 011 *B* = 0.828			*p* = 0.002 *B* = 0.564		*p* = 0.008 *B* = 0.947		*p* = 0.004 *B* = 1.015				
Hyoglossus	*p* = 0.053 *B* = − 0.482							*p* = 0.016 *B* = 0.689				
Age	*p* = 0.015 *B* = 0.301											
Tumour stage	*p* = 0.05 *B* = − 0.249									*p* = 0.007 *B* = − 0.382		
Node stage	*p* < 0.001 *B* = 0.517			*p* = 0.012 *B* = 0.334								

^*^B = Beta: co-efficient for each variable

### Summary of Findings

Most notably, increasing dosage to the IPC emerged as having a positive relationship with hyolaryngeal displacement and pharyngeal constriction during swallowing. This association was not seen for residue and time measures. Other patterns were also observed. Radiation dose to the cricopharyngeus was associated with reduced hyoid and laryngeal elevation and tongue base retraction. Radiation dose to the glottic larynx was associated with reduced hyoid and laryngeal elevation and pharyngeal constriction. Dosage to the base of tongue was associated with more residue in the valleculae and pyriform sinuses. Radiation dose to the genioglossus was associated with reduced processing time at all stages of the swallow. Female sex was associated with reduced elevation of the hypopharynx for the pudding thick texture. In general, more associations were observed for the thicker (pudding) consistency than the thin consistency. T and N stages were associated with pharyngeal constriction and valleculae residue, but no consistent patterns were observed. Statistical analysis results for each outcome variable are presented in Table 5.

## Discussion

This study aimed to investigate the relationship between dosimetry to key swallowing structures in IMRT and swallowing function in NPC survivors, using swallow kinematics as the primary method for evaluating swallow function. While previous studies have tended to focus on specific regions or geographies, this study covered a diverse range of swallowing-related structures and their kinematic and timing performance. This allowed for the examination of swallowing as a cohesive process with multiple inputs, rather than the actions of a few select muscles.

### Swallowing Kinematics in Healthy Adults and Head and Neck Cancer Survivors

In general, as expected, the kinematic measures of swallowing found in this study were lower than the figures reported in the literature for healthy adults while swallowing thin textures, including maximum hyoid displacement [17–20, 25] and hyolaryngeal approximation [20]. Kinematic measures in our NPC cohort more closely approximated that of healthy older adults [19, 25] and other dysphagic populations [26]. Other head and neck cancer studies that examined swallowing kinematics have also reported figures lower than healthy adult norms [27–30], but findings from different studies within this clinical population are difficult to compare due to differing methodologies in the volume and viscosity of the liquids swallowed during VFSS [27, 28], type of head and neck cancer [29], and the demographic and treatment history of the participants examined [30]. Timing measures of swallowing were not distinct in this cohort, with the findings falling within the wide range reported for healthy adults [31]. It is likely that NPC survivors (and the wider head and neck cancer survivor population) will present with performances across wide ranges for various swallowing kinematic measures, and these may depend on multiple variables in relation to treatment and the survivor’s individual traits [32].

### The Uniqueness of the Inferior Pharyngeal Constrictor

A major finding in the present study is the consistent emergence of the IPC and the nature of its relationship with hyolaryngeal displacement. While all the other muscles that were found to be associated with swallowing kinematics had a negative relationship, the relationships between the IPC and its relevant kinematic measures were consistently positive. This means that as radiation dosage increased to the IPC, hyolaryngeal displacement was better preserved. This is counterintuitive and is contrary to previous studies reporting that higher IPC dosimetry was associated with dysphagia [33, 34]. The explanation for this may be multifaceted. To measure dysphagia, Caglar and colleagues [33] used VFSS-confirmed aspiration and dysphagia severity, while Jiang and colleagues [34] used a patient reported swallowing questionnaire (MDADI), all of which are more gestalt measures of swallowing as compared to a single displacement measure. In addition, hyoid excursion magnitude can be comparable to normal healthy individuals despite the presence of dysphagia [35]. Therefore, the current findings may not be mutually exclusive from these two studies; rather, another perspective may be suggested based on the findings. Caglar and colleagues [33] and Jiang and colleagues [34] identified the IPC as key to dysphagia, but they did so without consideration of the suprahyoid muscles, which have since emerged as being potentially related to swallowing changes [13]. Indeed, the current study reflects this to a certain degree in the finding of the genioglossus as an associated structure with reduced processing time of the swallow. Additionally, Caglar and colleagues [33] did not focus on NPC survivors solely and included a range of head and neck cancer types in their study. Jiang and colleagues [34] focused on NPC and reported dosimetry of ≥ 47 Gy to be the cut off for predicting late dysphagia, which is exactly the mean dosage applied to the IPC in the cohort examined in this study (Table 3). Of course, differences in study design do not address the key issue of the nature of the relationship between the IPC and hyolaryngeal displacement reported here; rather, the distinctive histology of the IPC may offer more insight. The IPC has two layers of muscles, the inner layer is composed of type I muscle fibres predominantly, and the outer layer is composed mostly of type II muscle fibres [36]. Type IIB fibres have been shown to be more susceptible to radiation injury in animal models [37], therefore it may be speculated that the outer layer of the IPC becomes (more) injured in IMRT. This line of thinking has been raised previously to explain pharyngeal motility issues experienced by head and neck cancer survivors [38]. We would like to extend on this by further describing the inner layer of the IPC and nominate its function in conjunction with the suprahyoid muscles to elevate the hyolaryngeal complex as a logical explanation.

The demarcation of the pharyngeal constrictors in CT scans does not discriminate between the outer and inner layers of the IPC. The outer layer consists of the concentric constrictors that aid in the propulsion of the bolus through the pharynx, while the inner layer consists of three longitudinal muscles: palatopharyngeus, salpingopharyngeus, and stylopharyngeus. These muscles have been proposed to work in a two-sling action with the suprahyoid muscles to be the primary elevators of the hyolaryngeal complex [39, 40]. Following IMRT, as the outer layer of the constrictors are injured, the longitudinal muscles of the inner layer, predominantly type I fibres, may be better able to maintain their histological and physiological integrity. Coupled with the relatively lower dosage applied to the suprahyoid muscles in NPC, possibly due to absorption of radiation by soft tissue in the submandibular space [38], the elevation of the hyolaryngeal complex emerges as the measure that remains most preserved following radiation therapy. In conclusion, the positive relationship found between radiation dosage to the IPC and hyolaryngeal elevation does not indicate increased displacement of swallowing structures; rather, it is evidence of preservation of function due to the compensation effects of complementary muscle action.

### Swallowing-Related Structures and Kinematics

For four swallowing-related structures, a negative relationship was found between swallowing kinematics and radiation dosage: the cricopharyngeus, glottic larynx, base of tongue, and genioglossus. Radiation to the cricopharyngeus has been linked to increased risk of adverse outcomes including mortality in head and neck cancer patients [41, 42], including in studies that have adopted a comprehensive voxel-based dose analysis approach [42, 43]. The cricopharyngeus is also composed of slow-twitch type I muscle fibres, which means it should be more resistant to radiation injury. Its emergence as a key structure to swallowing kinematics is likely due to the relationship between UES opening, for which is it responsible, and the preceding and subsequent actions during swallowing [44]. The base of tongue was the only structure to demonstrate consistent links with increased post-swallow residue. Poor posterior tongue propulsion may lead to incomplete evacuation of the bolus to the oropharynx and pharynx, thus leaving the remainder of the bolus to spill into the valleculae and pyriform sinuses. Residue in the pyriform sinus may be additionally explained by poor pharyngeal constriction, as indicated by the association with radiation to the SPC (Table 5). However, the direction of this relationship requires further investigator as the findings suggest that higher dosage to the SPC is associated with reduced residue in the pyriform sinus. The findings of the glottic larynx and genioglossus as key structures is consistent with studies that reported the dose to larynx and suprahyoid muscles to be linked to dysphagia in mixed head and neck cancer types [45]. Female sex was associated with reduced hyolaryngeal elevation for the pudding thick texture, which points to sex differences that have been reported in swallowing kinematic studies [25]. Further studies with sex and age-matched control groups would yield additional insight into this issue, especially as occurrence of NPC tends to be higher in males, and females may be neglected in proactive management of the disease.

It should be noted that radiation dosimetry to specific sets of structures is only part of the overall picture of how swallowing function can be affected by IMRT. Radiation effects on soft tissues other than muscles, such as salivary glands [46] and sensory organs [47], can also contribute to the initiation and coordination of the swallow. The evidence and technologies we have thus far points towards minimisation of radiation to key swallowing structures as the approach with the most potential to target post-treatment dysphagia. The findings of this study contribute to the overall understanding of how radiation affects these structures, and has the potential to illuminate candidate structures for IMRT planning that maximises treatment effects while minimising damage to salient DARS.

## Limitations

The current study represents a cross-sectional long-term analysis of survivors of NPC treatment. NPC survivors who required tube-feeding were excluded, which may have limited the swallowing performance range. This study did not use clinical or functional indices of swallowing as outcome measures, which may yield different results. Changes in kinematics and timing may not reflect the functional integrity of the swallow. The current findings would be useful as reference for VFSS findings in NPC survivors, but should not be used alone as diagnostic criteria for dysfunction. While kinematic measures of swallowing can be reflective of functional swallowing outcomes in head and neck cancers [48], they would be best advised to be used in conjunction with validated and standardised swallowing assessment methods.

## Conclusion

Swallowing kinematics are negatively affected by radiation following IMRT with or without concurrent chemotherapy in NPC survivors at least 3 years post-treatment. Variables found to be key in this relationship were radiation dosage to the cricopharyngeus, glottic larynx, base of tongue, and genioglossus, and the female sex. The inferior pharyngeal constrictor was found to have a positive relationship with hyolaryngeal displacement, likely due to its unique muscular composition and the contribution of adjacent muscles to hyolaryngeal displacement during swallowing. Consideration of a comprehensive range of swallowing-related structures is recommended for future studies that seek to examine dose-response relationships. Further studies are indicated to explore the novel findings in more detail.
